# Challenges in diagnosis of isolated central nervous system vasculitis

**DOI:** 10.1002/brb3.12

**Published:** 2011-09

**Authors:** Amy W Amara, Khurram Bashir, Cheryl A Palmer, Harrison C Walker

**Affiliations:** 1 Department of Neurology, University of Alabama at BirminghamBirmingham, Alabama; 2 Departments of Pathology and Neurology, University of Alabama at BirminghamBirmingham, Alabama

**Keywords:** Vasculitis, hypereosinophilic syndrome, primary angiitis of the central nervous system, memory, aphasia

## Abstract

Isolated central nervous system (CNS) vasculitis is a rare and complicated disorder. Patients typically present with nonspecific neurologic symptoms such as headache and encephalopathy, and have variable progression and severity of the disease. Challenges to definitive diagnosis include the limitations of currently available diagnostic modalities with high likelihood of false-positive or false-negative findings. Imaging, serologic, and cerebrospinal fluid (CSF) evaluation, and even angiography can fail to establish the diagnosis. Often, brain biopsy is required. In order to illustrate these challenges, we report the case of a patient who presented with subacute cognitive decline and was ultimately diagnosed with isolated CNS eosinophilic vasculitis. Initial work-up included CSF and serologic analyses, magnetic resonance imaging (MRI), and cerebral angiography, but definitive diagnosis required brain biopsy. Immunosuppressive therapy resulted in clinical improvement and stabilization. To our knowledge, only one other case of isolated CNS eosinophilic vasculitis has been reported in the literature. We discuss the importance of a high index of clinical suspicion in cases of progressive nonspecific neurologic symptoms.

## Introduction

Central nervous system (CNS) vasculitis is a rare and diagnostically challenging disorder because patients present with nonspecific symptoms of variable severity and progression, such as headache and encephalopathy (Scolding 2009). Vasculitis isolated to the CNS is even less common than systemic vasculitis and may lack many of the diagnostic markers (i.e., elevated erythrocyte sedimentation rate) seen in the latter (Moore 1989; Panda et al. 2000). Additionally, isolated CNS vasculitis may mimic other disorders, such as infection, malignancy, or prion disease, which have overlapping symptomatology. Definitive diagnosis is further complicated because many diagnostic measures, including magnetic resonance imaging (MRI) and cerebral angiography can produce both false-positive and false-negative results for CNS vasculitis (Hankey 1991; Scolding 2009; Gomes). In many cases, brain biopsy is the only useful diagnostic study. However, even a negative brain biopsy does not rule out isolated CNS vasculitis (Chu et al. 1998; Birnbaum and Hellmann 2009). Based on published studies, the estimated sensitivity of cerebral angiography for detection of vasculitis is between 27% and 90% (Calabrese and Mallek 1988; Vollmer et al. 1993; Duna and Calabrese 1995; Chu et al. 1998; Alrawi et al. 1999; Salvarani et al. 2007; Birnbaum and Hellmann 2009) and that of brain biopsy between 36% and 83% (Calabrese and Mallek 1988; Lie 1992; Duna and Calabrese 1995; Chu et al. 1998; Salvarani et al. 2007; Birnbaum and Hellmann 2009). Thus, a high level of clinical suspicion is needed to make this elusive diagnosis. To illustrate these challenges, we report a case of a patient who presented with nonspecific symptoms and negative initial work up, in whom a brain biopsy was ultimately needed to diagnose isolated CNS eosinophilic vasculitis. This case serves to emphasize the need for aggressive work up in cases of suspected isolated CNS vasculitis, with willingness to pursue brain biopsy when the diagnosis is not clear.

## Presentation of Case

A 52-year-old right-handed woman presented to the emergency room with progressive short-term memory loss and word-finding difficulty. The symptoms began insidiously 3 months prior to her presentation to our institution with disorientation to person and place, impaired naming, and poor balance. Three weeks before admission, she worsened relatively rapidly with additional symptoms of personality change and comprehension difficulties. She denied any weakness or numbness, but complained of frontal headaches that she was unable to further characterize. Comprehensive review of symptoms was essentially negative, including no upper respiratory symptoms, fever, night sweats, arthralgias, or rash.

Her past medical history included hypertension, diabetes, hyperlipidemia, and chronic hearing loss. She did not have a history of migraine headaches or asthma. Her medications on admission included insulin glargine, pravastatin, benazepril, and metformin. The patient had previously worked in an office and denied any chemical or toxin exposures. She had a 40 pack-year history of smoking, having quit 20 years prior to presentation. There was no family history of cognitive deficit.

She was afebrile and had normal vital signs and general physical exam. On neurologic examination, she was awake and alert but oriented only to self. She was unable to name a watch or pen and could not follow multistep commands. There was no dysarthria, although she did display bradylalia. Cranial nerves were normal except for decreased hearing to finger rub bilaterally. Motor, sensory, and coordination exams were normal. Deep tendon reflexes were normal throughout with flexor plantar responses. Unstressed gait was narrow based with slightly unsteady tandem gait.

**Figure 1:**
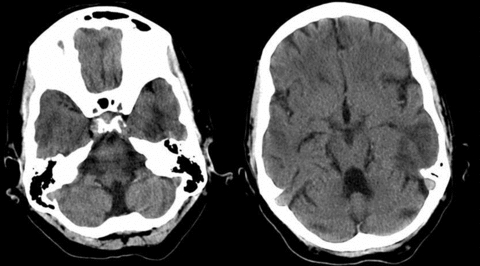
CT head upon presentation. Noncontrast CT of the head showing bilateral temporal hypodensities, left greater than right.

Noncontrast computed tomography (CT) of the head at presentation showed bilateral (left greater than right) temporal lobe hypodensities and diffuse atrophy (Fig. 1). The patient was admitted for further evaluation.

## Diagnosis

Based on the clinical presentation and imaging findings, the patient was empirically started on acyclovir in the emergency room, although the duration of her symptoms made Herpes encephalitis unlikely. The differential diagnosis for subacute cognitive decline is very broad and includes infections (human immunodeficiency virus [HIV], tuberculosis, neurosyphilis); primary CNS tumor including CNS lymphoma, or metastasis; multifocal infarcts; inflammatory/infiltrative processes such as sarcoidosis; vasculitis; demyelinating disease (progressive multifocal leukoencephalopathy, acute disseminated encephalomyelitis); neurodegenerative disease such as progressive dementia; prion disease; paraneoplastic limbic encephalitis; and exposures to toxins such as organic solvents. This initial broad differential is common in patients ultimately diagnosed with isolated CNS vasculitis secondary to the nonspecific neurologic symptoms.

**Figure 2:**
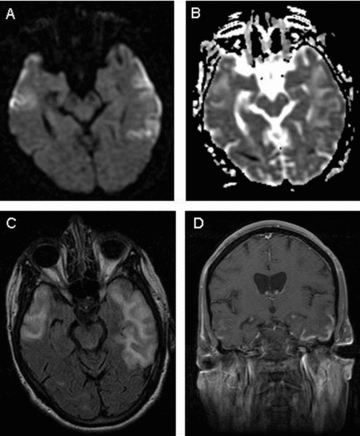
MRI brain. (A) Diffusion weighted image-revealing restriction of diffusion in gyriform pattern in bilateral temporal lobes, worse on the left, confirmed with apparent diffusion coefficient map (B). (C) FLAIR image showing hyperintesities in bilateral lateral temporal lobes. (D) Gyriform pattern of enhancement on postcontrast T1 image.

In this patient, an MRI of the brain with and without contrast showed T2/FLAIR hyperintense and T1 hypointense lesions in the bilateral lateral temporal lobes (left greater than right) with enhancement and restriction of diffusion in a gyriform pattern (Fig. 2). Magnetic Resonance Angiography (MRA) of the head and neck were normal.

Cerebrospinal fluid (CSF) examination showed 1 white blood cell, 148 red blood cells without xanthochromia, protein 61 mg/dL, and glucose 123 mg/dL. Herpes Simplex Virus (HSV) polymerase chain reaction (PCR), venereal disease research laboratory (VDRL), cryptococcal antigen, Gram stain, bacterial and fungal cultures, toxoplasma antigen, arbovirus PCR, West Nile Virus PCR, and varicella zoster PCR were all negative. Cytology and flow cytometry could not be performed on the CSF because there were too few cells. CSF and serum angiotensin converting enzyme levels were normal. Other normal or negative laboratory values included antinuclear antibody, erythrocyte sedimentation rate, cytomegalovirus IgG, rheumatoid factor, Sjogren's antibodies (SSA and SSB), cytoplasmic and perinuclear antineutrophil cytoplasmic antibodies (c-ANCA and p-ANCA), immunofixation electrophoresis, serum protein electrophoresis, urine protein electrophoresis, HIV, renal function, liver function, electrolytes, urine drug screen, and complete blood count with differential.

CT of the chest, abdomen, and pelvis demonstrated no evidence of lymphoma or primary malignancy. Long-term video EEG was normal. A four-vessel cerebral angiogram was normal without any evidence of vasculitis.

**Figure 3:**
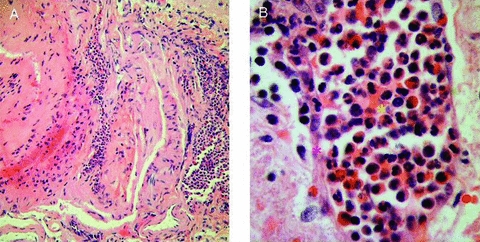
Brain biopsy. The vessel walls are obliterated by chronic inflammatory cells, the majority of which are eosinophils. Yellow Asterisk = vascular lumen; Red Asterisk = vessel wall. (A) H&E × 200. (B) H&E × 1000.

Because of the patient's continued symptoms and unclear diagnosis, the patient underwent a brain biopsy of the left anterior temporal lobe. Histopathological examination demonstrated a chronic astrogliosis of the gray matter without inflammation. The leptomeninges contained dilated vessels with neutrophils and eosinophils. Some vessel walls had been destroyed by the eosinophilic inflammation; both arteries and veins were involved. There were no granulomas or giant cells. Congo red staining did not show evidence of amyloid deposition in the vessels. These findings were consistent with eosinophilic vasculitis (Fig. 3). No parasitic or amebic organisms were seen. Review of her peripheral blood smear showed no peripheral eosinophilia.

## Treatment

The patient received high-dose glucocorticoid therapy with 1-g methylprednisolone intravenously once a day for 5 days. During that initial treatment, her language and memory improved, and she regained the ability to recognize and name family members. She also became oriented to person, place, and time, and her naming ability recovered. Her comprehension improved but she remained unable to perform complex tasks. She was subsequently placed on a prolonged oral prednisone taper and continued to have some mild additional improvement in cognition over the next month. The patient was then started on oral cyclophosphamide (2 mg/kg) and her symptoms have remained stable through follow-up over 2 years. She was monitored closely for bone marrow suppression associated with cyclophosphamide. After 2 years of cyclophosphamide administration, she developed microscopic hematuria despite aggressive hydration. A cystoscopy was negative for transitional cell carcinoma. Oral cyclophosphamide therapy was discontinued and she has continued to remain clinically stable.

## Discussion

This case serves to illustrate the diagnostic challenges of isolated CNS vasculitis. This entity is difficult to define clinically because its presentation is so variable. Vasculitis must therefore remain in the differential diagnosis in cases with nonspecific neurologic decline where no other etiology is apparent. In this case, brain biopsy was required to make the diagnosis. The decision to pursue brain biopsy should be considered when the diagnosis is not clear. Angiography may appear normal, as was the case in our patient, even in the presence of biopsy or autopsy proven CNS vasculitis (Alrawi et al. 1999). Alternatively, other conditions such as vasospasm or atherosclerosis (Duna and Calabrese 1995) may mimic vasculitis on a cerebral angiogram. In fact, the estimated specificity of angiography for detecting vasculitis is between 14% and 60% (Chu et al. 1998; Kadkhodayan et al. 2004) and that of MRI is between 19% and 36% (Calabrese and Duna 1995; Duna and Calabrese 1995; Chu et al. 1998), while that of brain biopsy is between 87% and 100% (Duna and Calabrese 1995; Chu et al. 1998). An additional role for biopsy in these cases is to rule out alternative diagnoses, such as CNS lymphoma or infection, for which incorrect or delayed treatment could lead to poor outcome. The most commonly encountered risk associated with stereotactic brain biopsy is hemorrhage, the incidence of which ranges from 8 to 9%, with 1–4% of these hemorrhages being symptomatic (Field et al. 2001; McGirt et al. 2005). Overall mortality and morbidity have been estimated at 0.7% and 3.5%, respectively (Hall 1998). In each case, the risk of brain biopsy should be weighed against potential for incorrect diagnosis or continued/progressive neurologic disability.

The etiology of this particular case of isolated CNS eosinophilic vasculitis is unclear. The patient did not have peripheral eosinophilia as would be expected if the vasculitis were secondary to a hypersensitivity allergic reaction or parasitic infection and there were no parasites or amebae seen on brain biopsy. Hypereosinophilic syndrome (HES), defined as blood and/or tissue eosinophilia without underlying allergic, parasitic, or other cause (Sheikh and Weller 2009) is a possible diagnosis. Neurologic manifestations in combination with systemic disease are common in HESs (∼50%) (Sheikh and Weller, 2007). Churg-Strauss syndrome (CSS) is an HES associated with peripheral eosinophilic vasculitis that may also be associated with neurologic symptoms (∼60%) (Sehgal et al. 1995). However, diagnosis of CSS requires four of the following six diagnostic criteria: asthma, peripheral eosinophilia >10%, mono- or polyneuropathy, pulmonary infiltrates, paranasal sinus abnormality, and extravascular eosinophils (Masi et al. 1990). Our patient did not meet these criteria. Additionally, CSS is characterized by granulomatous eosinophilic vasculitis, and granulomata were absent in the biopsy specimen obtained from our patient. There have been case reports of “limited” CSS with eosinophilic vasculitis or extravascular granulomas in the absence of blood eosinophilia or asthma. These cases have included vasculitic involvement of the skin and eyes (Khan et al. 1996), lungs (Sevinc et al. 2004), kidneys (Sharma et al. 2004), and heart (Taira et al. 2005). There has also been one previous report of isolated CNS eosinophilic vasculitis in the absence of asthma or peripheral eosinophilia (Sommerville et al. 2007). As in our case, the patient reported by Sommerville et al. had absence of granulomata or amyloid on biopsy and imaging findings were similar in the two cases. Both cases required brain biopsy for definitive diagnosis (Sommerville et al. 2007), suggesting that isolated CNS eosinophilic vasculitis may be an underrecognized entity within the spectrum of HESs.

An alternative diagnosis is primary angiitis of the CNS (PACNS). The diagnostic criteria of this entity include: (1) unexplained neurologic deficit after thorough clinical and laboratory evaluation; (2) evidence of an arteritic process by cerebral angiography and/or tissue examination; and (3) no evidence of a systemic vasculitis or any other condition to which the angiographic or pathologic findings could be secondary (Calabrese and Mallek 1988). The presence of eosinophils is unusual for this condition, arguing against classification of this case as definite PACNS.

In summary, this case of isolated CNS eosinophilic vasculitis demonstrates the difficulty encountered in establishing a diagnosis in cases of isolated CNS vasculitis in patients with subacute cognitive decline. Despite extensive laboratory, imaging, and angiographic evaluation, diagnosis often requires brain biopsy. This potentially neurologically devastating disorder is treatable with immunosuppressant therapy and therefore definitive diagnosis is critical. A relatively high index of suspicion and willingness to pursue a brain biopsy is often necessary to diagnose isolated CNS vasculitis.
